# Water quality assessment based on multivariate statistics and water quality index of a strategic river in the Brazilian Atlantic Forest

**DOI:** 10.1038/s41598-020-78563-0

**Published:** 2020-12-16

**Authors:** David de Andrade Costa, José Paulo Soares de Azevedo, Marco Aurélio dos Santos, Rafaela dos Santos Facchetti Vinhaes Assumpção

**Affiliations:** 1 Federal University of Rio de Janeiro (UFRJ), Alberto Luiz Coimbra Institute for Graduate Studies and Engineering Research (COPPE), Centro Tecnológico, Cidade Universitária, Rio de Janeiro, RJ Brazil; 2 Federal Fluminense Institute, São João da Barra Advanced Campus, BR 356, KM 181, São João da Barra, RJ Brazil; 3 Oswaldo Cruz Foundation (Fiocruz), National School of Public Health Sergio Arouca (ENSP), Rua Leopoldo Bulhões, 1.480, Manguinhos, Rio de Janeiro, RJ Brazil

**Keywords:** Environmental sciences, Limnology

## Abstract

Fifty-four water samples were collected between July and December 2019 at nine monitoring stations and fifteen parameters were analysed to provide an updated diagnosis of the Piabanha River water quality. Further, forty years of monitoring were analysed, including government data and previous research projects. A georeferenced database was also built containing water management data. The Water Quality Index from the National Sanitation Foundation (WQI_NSF_) was calculated using two datasets and showed an improvement in overall water quality, despite still presenting systematic violations to Brazilian standards. Principal components analysis (PCA) showed the most contributing parameters to water quality and enabled its association with the main pollution sources identified in the geodatabase. PCA showed that sewage discharge is still the main pollution source. The cluster analysis (CA) made possible to recommend the monitoring network optimization, thereby enabling the expansion of the monitoring to other rivers. Finally, the diagnosis provided by this research establishes the first step towards the Framing of water resources according to their intended uses, as established by the Brazilian National Water Resources Policy.

## Introduction

Aquatic systems have been significantly affected by human activities causing water quality deterioration, decreasing water availability and reducing the carrying capacity of aquatic life^1–4^. Water quality deterioration still persists in developed countries, while it is a major problem in developing countries in which a substantial amount of sewage is discharged directly into rivers^5–8^. Moreover, according to UNEP^9^, water pollution has worsened since the 1990s in the majority of rivers in Latin America. The global concern with water availability and its quality has been growing, and it is estimated that the demand for water will increase between 20 and 30% by 2050^10,11^. In addition, spatial and temporal variations in the hydrological cycle and their uncertainties related to climate change may worsen this scenario^12–16^.

Monitoring water quality in order to assess its spatial and temporal variations is essential for water management and pollution control^17^. On the other hand, monitoring programs generate large data sets that require interpretation techniques^18^. There are a number of methods for water quality assessment, including single-factor, multi-index, fuzzy mathematics, grey system evaluation, artificial neural network, multi-criteria analysis, geographical interpolation and multivariate statistical approach^18–25^. Among them, the most used are the Water Quality Indexes (WQI) that transform a complex set of data into a single value indicative of water quality^26,27^ and reflect its suitability for different uses^28^. Multivariate statistics is another widely used approach^29,30^, mainly with Principal Components Analysis (PCA) and Cluster Analysis (CA), helping to achieve a better understanding of the spatial and temporal dynamics of water quality.

A comparison of seven methods for assessing water quality indicated WQI as one of the best^20^. The assessment of Poyang Lake^28^, China and the upper Selenga River^31^, Mongolia showed that WQIs are suitable for the assessment of both interannual trends and seasonal variations^28^. Multivariate statistical techniques associated with WQI have been used for numerous water bodies world-wide, including the Nag River^30^, India, the Paraíba do Sul River^32^, Brazil, and the before mentioned Selenga River^31^. CA grouped the monitoring stations according to their similarities, while the PCA highlighted components that were related to its pollution sources^30–32^.

In order to ensure water quantity and quality, the Brazilian National Water Resources Policy^33^ has established a management tool called Framework, according to the main intended uses of water. It has also created participatory management committees, the so-called Basin Committees, which, together with its technical agency, are responsible for the Framework establishment. Unfortunately, even after two decades, Brazil has had very few successful experiences on the subject^34^.

Brazil has a gigantic and complex hydrographic network present in many different ecosystems^34^. The Brazilian Atlantic Forest is one of the most biodiverse biomes on the planet^35,36^, extending along the Brazilian coast and currently covering only 11.4% of its original territory^37^ under constant threats^38–40^. The hydrographic basin of the Paraíba do Sul river is located in this environment, which is the integration axis of the most industrialized Brazilian states, São Paulo, Rio de Janeiro and Minas Gerais, and home to around 6.2 million people^41^. A water transfer system regularly supplies another 9 million people in the metropolitan region of Rio de Janeiro, through the Guandu system. Another water transfer system connects the Paraíba do Sul river to the Cantareira system, complementing with 5 m^3^/s the water supply to over 9 million people in the metropolitan region of São Paulo^41^. These systems went through an intense water scarcity between 2014 and 2016 with severe impacts on water quality and availability^32^.

Our study is focused on the Piabanha River watershed, a strategic sub-basin of the Paraíba do Sul river, combining urban, industrial, rural characteristics, and large preserved fragments of Atlantic Forest^36,42^. The Piabanha Basin has been monitored for over 10 years with the Studies in Experimental and Representative Watersheds (EIBEX) project, a partnership between universities and government agencies^42–44^. The State Environmental Agency of Rio de Janeiro (INEA) has been monitoring the basin since 1980. Other studies in the region include the analysis of contamination by pesticides^45^, energy generation^46^ and dispersion of pollutants^47^. The Piabanha Basin received international attention in Nature's article on biodiversity^36^. But in addition to forest preservation, can the Piabanha River support biodiversity? How is its water quality today? In this way, the Piabanha Basin Committee defined the Framework as a priority in its management plan (2018–2020) and to accomplish this goal, established water monitoring as a strategic action^48^.

Our study covers 40 years of monitoring, including government data, our research projects and, currently, a monitoring program that is being conducted with funding from the Piabanha Basin Committee. The main objectives were: (1) to carry out an updated diagnosis of water quality using multivariate techniques and WQI; (2) to examine the parameters that most influence water quality, and (3) to identify river stretches with similar water quality. Our study provides an extensive understanding of the Piabanha River and supports its Steering Committee in the application of public policies. This is a pilot project that can be a reference for other Framework programs for improving water quality in Brazil.

## Results

### Water uses

We have requested and received from INEA two water user databases of the Piabanha Basin. The first set corresponds to raw data from the National Water Resources Register (CNARH), with all the registrations until December 2017 and with 1549 registered interferences (water abstraction or effluent discharge). The second one is the registration validated by INEA until August 2018 by the Águas do Rio project comprising a total of 669 validated interferences. With these data, it was possible to build a georeferenced base. By so doing, it was possible to list the main effluent discharges by type for each monitoring station.

In the validated database, from the 669 interferences, 84% are water abstractions and 16% are effluent discharges. Water abstraction account for 425 m^3^ day^−1^ with 75% from wells and 25% from rivers. On the other hand, effluent discharges are 89 m^3^ day^−1^. The largest volume of effluents comes from the sanitation sector with 57% of the total, whereas industries account for 33%, aquaculture with 4% and mining for 3% of discharges.

When comparing the two databases, it is clear that the universe of registered users is much larger than the universe of validated users; in other words, those whose data were made up by the state environmental agency and, therefore, received a license. For example, the validated database has only six interferences related to agriculture, in contrast to 789 interferences awaiting validation. This is a serious obstacle for water resources management in the region, which threatens the sustainability of water resources.

### Short time monitoring and water quality index

**Figure 1:**
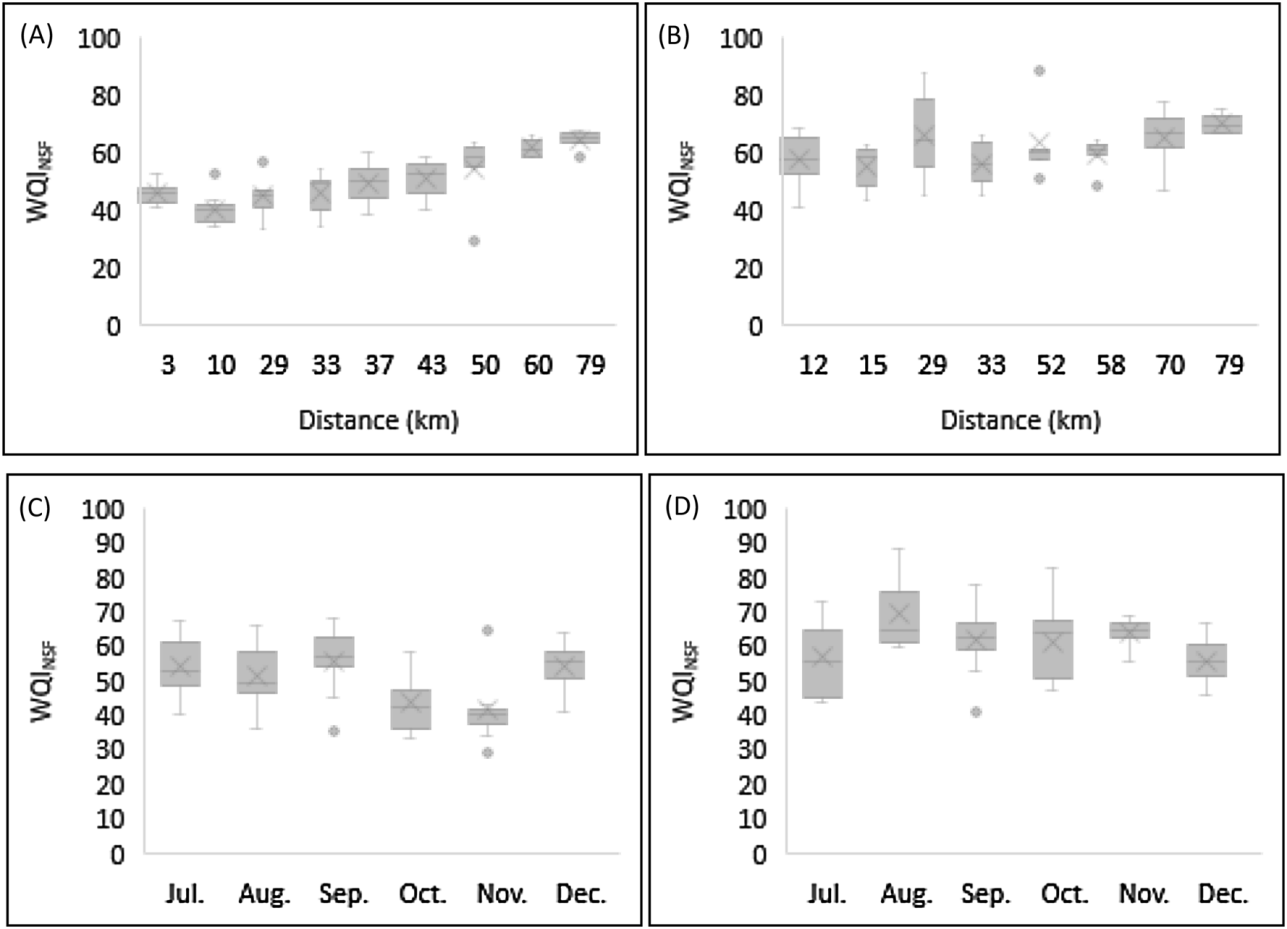
WQI_NSF_ spatial variation over each station from July to December (**A**) 2012 and (**B**) 2019. WQI_NSF_ seasonal variation over the entire length of the river (**C**) 2012 and (**D**) 2019. The entire dataset can be found online as Supplementary Table S1 and S2, respectively for 2012 and 2019.

In order to assess and compare the water quality of the Piabanha River, we calculated the Water Quality Index from the National Sanitation Foundation (WQI_NSF_) using two datasets, the first one from 2012 and the last one from 2019 (Tables 1 and Table 2). The 2012 results (Fig. 1A) oscillated between the bad and medium categories, generally with medium quality (50.5 ± 10.3). In 2019 (Fig. 1B), the results ranged between the medium and good categories, in general with medium quality (61.6 ± 10.8).

**Table 1:** Parameters, abbreviations, units, quantification limits, permissible limits to rivers class 2 and methods. Field measurements were performed using a multiparameter probe (YSI model 556) and a portable turbidimeter (HANNA model HI 98703-0). Laboratory analyses follows the Standard Methods for the Examination of Water and Wastewater (SMWW).

Parameters	Abbreviation	Units	Quantification limits	Permissible limits—class 2	Method
Electrical conductivity	EC	µS/cm	0.01	–	Field measure
Water temperature	Temp	°C	0.1	–	Field measure
Turbidity	Turb	NTU	0.01	100	Field measure
Dissolved oxygen	DO	Mg L^−1^	0.01	> 5	Field measure
pH	pH	pH units	0.01	6–9	Field measure
Total dissolved solids	TDS	mg L^−1^	10	500	SMWW 2540C
Suspended solids	SS	mg L^−1^	1	–	SMWW 2540D
Alcalinity	Alcal	mg/L (CaCO_3_)	3	–	SMWW 2320B
Biological oxygen demand	BOD	mg L^−1^	2	5	SMWW 5210B
Chemical oxygen demand	COD	mg L^−1^	10	–	SMWW 5220D
*Escherichia coli*	*E. coli*	CFU/100 mL	1	1000	SMWW 9223A/B
Phosphate	PO_4_^3−^	mg L^−1^	0.02	–	SMWW 4500 P E
Total phosphorus	TP	mg L^−1^	0.02	0.1	SMWW 4500 P E
Nitrate	NO_3_^-^	mg L^−1^	1	10.0	SMWW 4500D
Ammonium	NH_3_	mg L^−1^	0.06	3.7 (pH ≤ 7.5)	SMWW 4500F
Total nitrogen (Kjeldahl)	TN	mg L^−1^	2	2.18	SMWW 4500A

**Table 2:** Average seasonal results in 2019. Distances refers to measures from the source to the mouth of the Piabanha River. Station 9 is located on the Paquequer/Preto river. Abbreviations, units, methods, quantification limits and permissible limits to rivers class 2 are described in Table 1. The entire dataset can be found online as Supplementary Table S2.

Dist (km)	Station	Season	DO	Water temp	*E. coli*	pH	BOD	NO_3_^−^	PO_4_^3−^	Turb	TDS	WQI_NSF_	SS	Alcal	COD	TP	NH_3_	TN
12	1	Dry	7.71	21.04	7467	7.26	22.67	1.00	0.58	4.72	173	52.03	26	67	59.33	0.83	8.00	8.97
		Wet	8.72	21.79	3673	6.73	3.33	1.00	0.43	5.85	158	62.77	10	57	16.33	0.74	5.92	6.73
15	2	Dry	7.83	20.42	67,867	7.38	10.67	1.16	0.83	8.26	167	55.77	14	77	31.00	1.21	9.75	12.00
		Wet	5.84	22.07	16,327	6.79	8.33	1.02	0.61	5.64	168	54.03	11	60	28.00	0.99	6.00	8.33
29	3	Dry	7.70	20.60	67,100	7.15	5.33	1.16	0.51	5.05	138	65.60	7	46	14.67	0.78	6.43	8.33
		Wet	6.47	21.87	7800	6.91	6.33	1.00	0.33	5.06	134	66.63	10	47	25.67	0.56	4.75	6.00
33	4	Dry	8.13	19.75	9500	7.40	13.00	1.05	0.41	10.47	118	52.63	26	45	36.33	0.63	4.53	6.53
		Wet	5.76	23.06	9007	7.01	5.67	1.00	0.19	13.31	96	60.17	15	38	25.33	0.41	2.67	4.23
52	5	Dry	8.31	19.31	3834	7.22	7.00	3.97	0.42	6.32	135	68.90	10	33	21.67	0.65	1.72	7.53
		Wet	6.69	24.29	10,367	7.02	7.00	2.22	0.18	34.93	86	57.37	41	20	30.67	0.44	1.01	5.27
58	6	Dry	8.01	19.90	4467	7.93	3.33	2.92	0.16	10.77	84	62.30	8	21	12.33	0.31	0.53	4.83
		Wet	6.84	24.89	5100	6.95	7.67	1.72	0.09	62.47	73	56.80	48	18	29.00	0.21	0.41	2.70
70	7	Dry	7.52	20.84	714	7.39	3.67	3.42	0.16	11.82	91	72.10	10	20	13.33	0.28	0.19	5.07
		Wet	6.75	24.59	4490	7.06	6.33	2.07	0.07	145.20	65	58.27	69	17	29.67	0.24	0.11	3.23
79	8	Dry	7.72	20.41	270	7.39	3.33	3.23	0.15	9.44	90	73.27	7	22	11.33	0.26	0.08	3.70
		Wet	6.81	24.59	627	7.07	5.67	2.23	0.09	45.60	64	66.93	32	18	22.67	0.25	0.11	4.00
–	9	Dry	9.04	17.75	1113	7.33	4.00	3.25	0.14	6.54	105	68.53	8	25	14.33	0.33	1.32	6.57
		Wet	5.91	22.37	10,967	7.15	15.00	2.01	0.05	34.53	72	51.63	51	22	48.33	0.30	0.67	4.23
Mean (n = 54)			7.32	21.64	12,816	7.17	7.69	1.97	0.30	23.67	112	61.43	22	36	26.11	0.52	3.01	6.01
Standard deviation			1.61	2.79	37,759	0.60	7.52	1.12	0.24	49.47	47	11.02	30	21	21.65	0.33	3.13	2.53
Maximum			11.72	26.12	200,000	8.75	45.00	4.44	0.93	330.00	248	88.10	147	91	114.00	1.65	10.00	13.00
Minimum			3.40	15.59	1	6.11	2.00	1.00	0.03	3.58	43	33.70	1	10	10.00	0.15	0.06	2.00

**Table 3:** Pearson correlation (r). Correlation values are given below the main diagonal of the matrix. The p-values (two-tailed probabilities that the variables are uncorrelated) are given above the main diagonal of the matrix.

r/p	DO	WT	*E. coli*	pH	BOD	NO_3_^−^	PO_4_^3−^	Turb	TDS	SS	Alcal	COD	TP	NH_3_	TN
DO		0.93	0.22	0.62	0.26	0.89	0.95	0.78	0.64	0.83	0.87	0.27	0.94	0.90	0.98
WT	0.03		0.05	0.05	0.06	0.01	< 0.01	< 0.01	< 0.01	< 0.01	< 0.01	0.10	< 0.01	< 0.01	0.01
*E. coli*	− 0.49	− 0.70		0.22	0.71	0.12	0.02	0.19	0.07	0.03	0.06	0.88	0.03	0.04	0.02
pH	− 0.21	0.72	− 0.49		0.14	0.21	0.04	0.15	0.01	0.11	0.04	0.18	0.04	0.03	0.03
BOD	0.45	− 0.69	0.16	− 0.57		0.06	0.05	0.13	0.02	0.31	0.01	< 0.01	0.05	0.03	0.07
NO_3_^−^	0.06	0.82	− 0.60	0.50	− 0.68		0.04	0.07	0.03	0.04	< 0.01	0.13	0.05	< 0.01	0.09
PO_4_^3−^	− 0.03	− 0.86	0.77	− 0.73	0.71	− 0.72		0.05	< 0.01	0.03	< 0.01	0.09	0.00	< 0.01	< 0.01
Turb	− 0.12	0.87	− 0.52	0.55	− 0.59	0.67	− 0.70		0.04	< 0.01	0.04	0.23	0.05	0.04	0.07
TDS	0.19	− 0.87	0.67	− 0.81	0.80	− 0.75	0.97	− 0.72		0.03	< 0.01	0.04	< 0.01	< 0.01	< 0.01
SS	0.09	0.85	− 0.75	0.61	− 0.41	0.74	− 0.75	0.91	− 0.74		0.03	0.52	0.04	0.02	0.05
Alcal	0.07	− 0.87	0.68	− 0.73	0.81	− 0.85	0.96	− 0.72	0.97	− 0.76		0.04	< 0.01	< 0.01	< 0.01
COD	0.44	− 0.62	0.06	− 0.53	0.98	− 0.59	0.64	− 0.48	0.72	− 0.27	0.73		0.09	0.07	0.11
TP	− 0.03	− 0.86	0.77	− 0.73	0.70	− 0.70	1.00	− 0.70	0.96	− 0.73	0.95	0.64		< 0.01	< 0.01
NH_3_	0.06	− 0.89	0.73	− 0.75	0.77	− 0.87	0.96	− 0.73	0.97	− 0.80	0.99	0.67	0.94		< 0.01
TN	− 0.01	− 0.83	0.77	− 0.76	0.67	− 0.64	0.99	− 0.66	0.96	− 0.71	0.93	0.60	0.99	0.92

**Table 4:** PCA loadings, values greater than 0.50 or less than -0.50 are very significant.

	DO	WT	*E. coli*	pH	BOD	NO_3_^−^	PO_4_^3−^	Turb	TDS	SS	Alcal	COD	TP	NH_3_	TN
PC 1	0.07	− 0.94	0.72	− 0.78	0.78	− 0.83	0.97	− 0.80	0.98	− 0.82	0.98	0.70	0.96	0.99	0.94
PC 2	0.87	0.09	− 0.66	− 0.10	0.58	0.05	− 0.09	0.04	0.10	0.33	0.04	0.64	− 0.09	− 0.02	− 0.09

Data sets show significant seasonal behavior (p < 0.05) (Fig. 1C,D) between the end of the dry period (Jul, Aug, Sep) and the beginning of the rainy period (Oct, Nov, Dec) for the parameters DO, WT, pH, nitrate, phosphate and turbidity, while no significant seasonal difference (p > 0.05) was found for the parameters *E. coli*, BOD and TDS. The parameters that have most impacted the WQI_NSF_ were coliforms and BOD. Ammonia and total phosphorus do not account to WQI_NSF_, but their concentration has violated Brazilian legislation and their influence can be better understood by PCA.

### Principal components and clusters analysis

The 2019 dataset (n = 48), comprising six monitoring campaigns at the eight monitoring stations along the Piabanha River with 15 parameters analysed, was grouped by the average value of each parameter at each station (n = 8). Pearson’s correlation matrix is presented in Table 3, most parameters showing a strong correlation (r > 0.5) with a confidence interval greater than 95% (α = 0.05). The KMO measures of sampling adequacy (n = 8) were near to 0.5 and the significance level of test of sphericity was less than 0.001, indicating that the data was fit for PCA and the correlation matrix is not an identity matrix and so the variables are significantly related. The Shapiro test confirmed the data normality (p > 0.01) for all parameters, except for *E. coli*.

**Figure 2:**
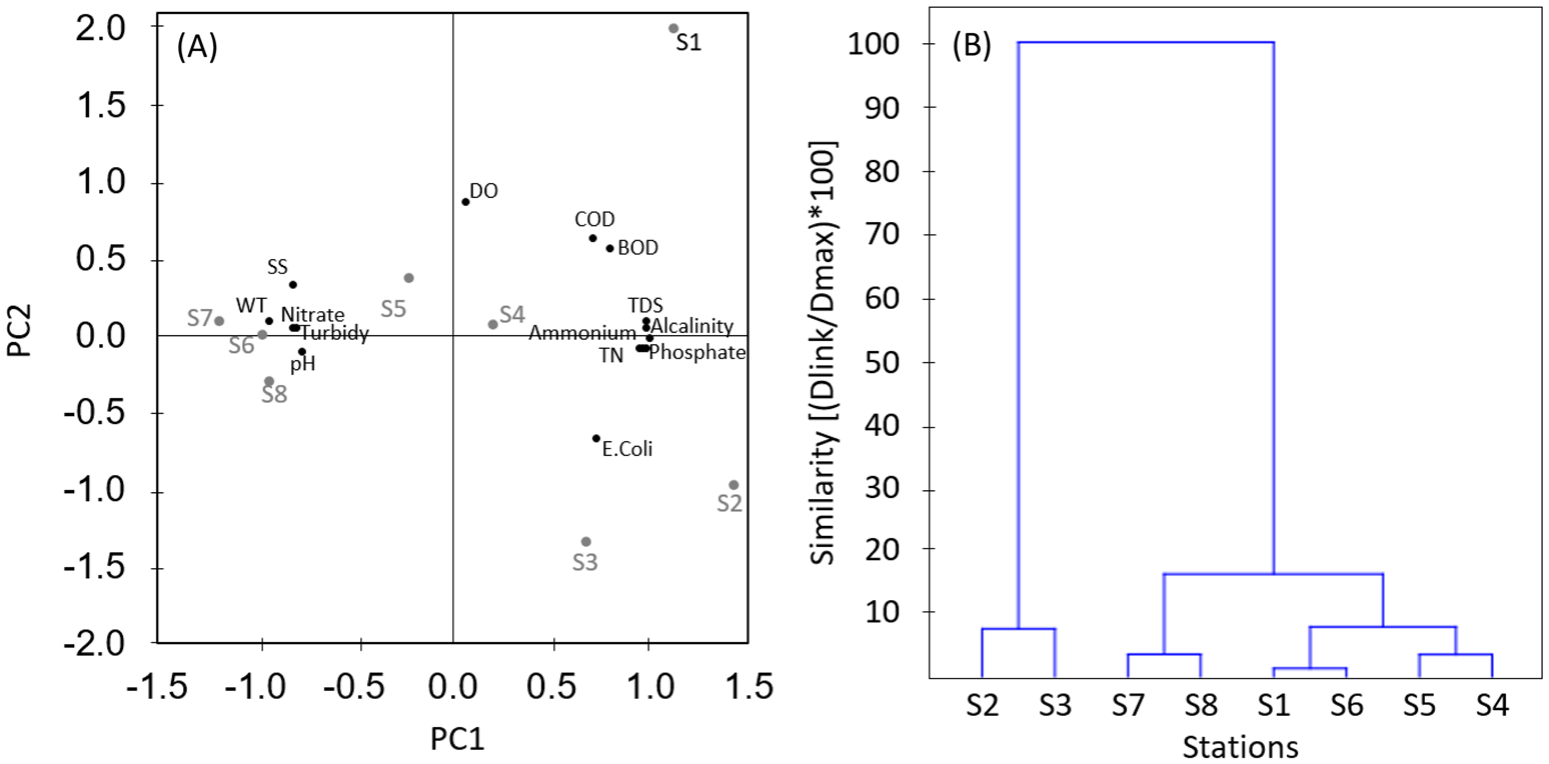
Multivariate techniques. (**A**) PCA plot with station scores and parameters loadings. (**B**) Hierarchical clustering by Ward linkage with Euclidean distance. The entire dataset can be found as Supplementary Table S2 online.

ACP was applied to identify groups of parameters that influence water quality. PC 1, PC 2 and PC3 account for 72% (eigenvalue 10.74), 14% (eigenvalue 13.94) and 5% (eigenvalue 0.8), respectively, of the data variance. Components with eigenvalues larger than the unit were selected. That is, the first two components together account for 86% of the total variance. The loadings that compose the first two components are presented in the Table 4 and the stations that most influence the results are represented in Fig. 2A.

PC1 was substantially correlated with practically all parameters. Stations number 1 to 4 loaded positively (loadings > 0.7) to PC1 with the parameters TDS, Alkalinity, Ammonia, Total Nitrogen, Phosphate, Total Phosphorus, DBO, COD, *E. coli*, while stations number 5 to 8 loaded negatively (loadings < − 0.7) with Nitrate, Turbidity, SS, pH and WT. PC2 was most influenced by stations in the urban area, notably station 1, and showed a positive correlation (loadings > 0.5) with OD, COD, BOD and less by SS (loading = 0.33), being more influenced by station 1 in the urban area. On the other hand, it was negatively correlated with *E. coli* (loading = − 0.66) with a large influence of station 3.

The sampling stations were grouped into three statistically significant clusters with 75% of similarity by agglomerative hierarchical clusterization based on the ward linkage by Euclidean distance (Fig. 2B): cluster 1 (Stations 2 and 3), cluster 2 (Stations 7 and 8) and cluster 3 (Stations 1, 4, 5 and 6).

### Longtime monitoring assessment based on Mann–Kendall rank test and Fourier transform

In a complementary way, in order to evaluate a possible trend on water quality and to detect the seasonal behavior of the basin, we used a time series with 40 years of monitoring. Since dissolved oxygen can be used as a surrogate variable for the general health of aquatic ecosystems^49–51^, it was selected to perform the Mann–Kendall rank test of randomness for the station more upstream and further downstream of the Piabanha River, PB002 and PB011 respectively. The upstream station showed a statistically significant increasing trend (n = 166, S = 1507, Z = 2.10, p < 0.03), whereas the downstream station does not show a statistically significant trend (n = 198, S = 1179, Z = 1.27, p = 0.20). The entire dataset can be found as Supplementary Table S3 and S4.

**Figure 3:**
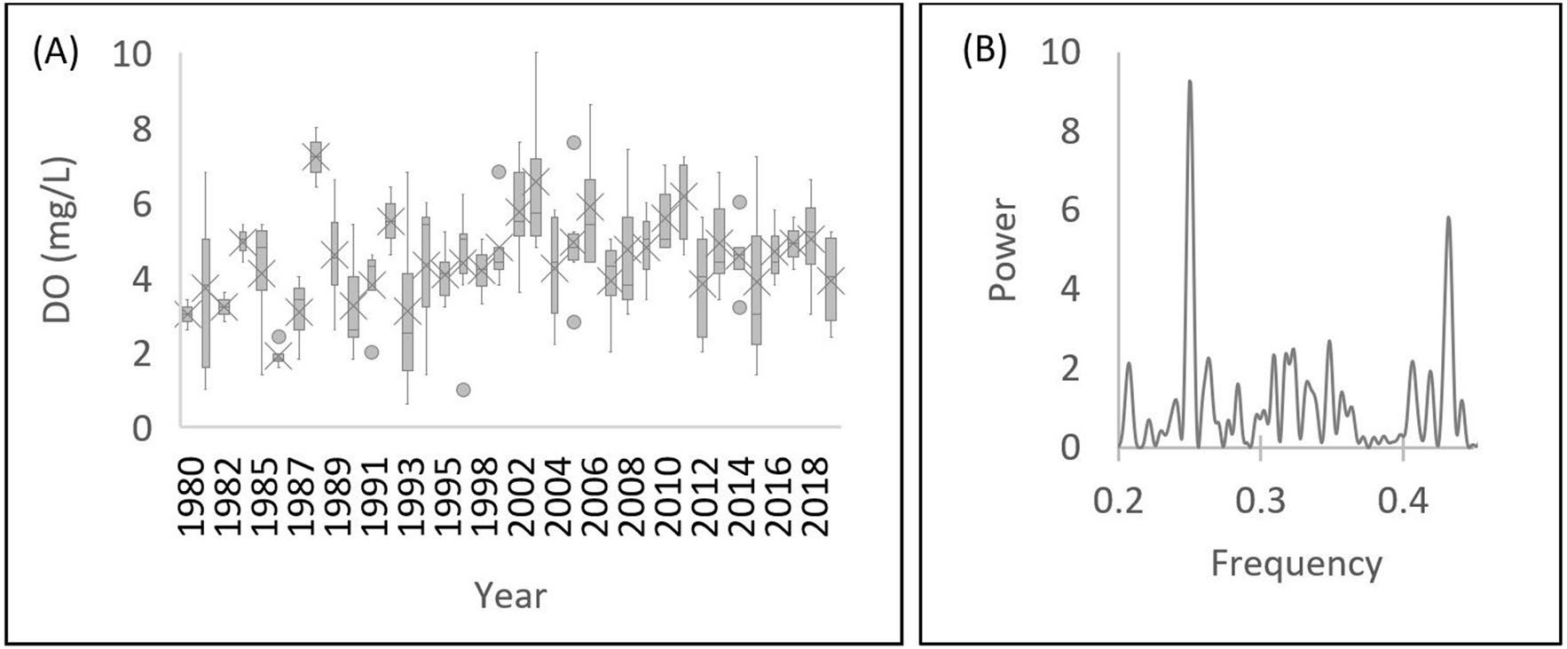
(**A**) Temporal distribution of dissolved oxygen from 1980 to 2019 at station PB002 (n = 160). (**B**) Periodogram. The entire dataset can be found in Supplementary Table S5.

To detect the seasonal behavior, we have applied a Fourier transform algorithm to the time series from 1980 to 2019 to the station PB011 (Fig. 3A, which does not display a tendency behavior and can be considered as representative of the entire basin because it is the most downstream station. The data were organized in quarterly averages for the DO parameter. The two most powerful signals correspond to the frequencies of 0.25 and 0.45, nearly (Fig. 3B) It corresponds to periods of 12 and 6 months, respectively. Taking into account this seasonality, we confirmed that our 2019 field campaigns are representative of seasonality comprising the final half of the dry season and the initial half of the rainy season.

## Discussion

### Water quality assessment

The Piabanha River had a better water quality in 2019 than in 2012, according to WQI_NSF_ results (Fig. 1). The improvement was substantial over the first 40 km, rated as “bad” in most campaigns in 2012, while rated as medium in most campaigns in 2019 due to sewage collection and treatment system expansion. Since 2012, Petrópolis has built 50 km of sewage collection network and 7 new sewage treatment units^52^. These plants produce secondary level effluents through biological treatment, the plants flow capacity reaches about 800 L s^−1^. These stations use different technologies such as: submerged aerated biofilters, anaerobic upflow reactor, moving bed biofilm reactor and upflow anaerobic sludge blanket reactor. Beside this, in some plants are used biosystems^53^. Water quality improved in stretches after 40 km due to self-purification processes and the contribution of clean tributaries. This is in line with findings from other rivers worldwide^31,54,55^.

Dry seasons, in general, presented better water quality indexes than rainy seasons. Other studies^28,56,57^ have shown similar seasonal behavior, where water quality worsens in the rainy season due to sediments and pollutants input carried by the rain. In addition, most of the sewage network is the same network that collects rainwater. Thus, during rainy events, sewage is no longer treated and is discharged directly into rivers.

Although the WQI_NSF_ had a medium rating in 2019, BOD and Coliforms were substantially above the maximum allowed by Brazilian regulation. In addition, the index is limited to the parameters used in its calculation^58^. This is the case for the ammonium parameter, which presented concentrations up to three times higher than allowed in Brazilian regulation, reminding that only nitrate is used in the WQI_NSF_. The same occurs with total phosphorus: only phosphate is considered, although it does not have a maximum value established by the Brazilian federal regulation. In what follows, we analyse these parameters in more detail.

Biochemical Oxygen Demand (BOD) is one of the most widely used criteria for water quality assessment. It provides information on the ready biodegradable fraction of the organic load in water^59^. High BOD concentrations reduce oxygen availability, mainly correlated to microbiological activity^60^. Its concentration ranged from 2.00 to 45 mg L^−1^ (average 7.69 ± 7.52) over the entire data, with its concentrations most of the time substantially above the maximum allowed by Brazilian regulation (5 mg L^−1^). *Escherichia coli* is naturally present in the intestinal tracts of warm-blooded animals and it is widely used as an indicator of fecal contamination^61,62^. Villas-Boas^42^ pointed to fecal coliforms as the most relevant water quality parameter in the urban area of Petrópolis, mainly related to pollution caused by untreated domestic sewage.

Phosphorus is an essential nutrient for all forms of life^63^. Its availability can be related to atmospheric deposition^64^, anthropic uses of products such as detergents^65^ and due to agricultural activities^66^. Orthophosphates are the most relevant in the aquatic environment as they are the main form of phosphate assimilated by aquatic vegetables^67^. Previous studies^42,68,69^ in the Piabanha Basin found phosphate values in perfect agreement with ours. Alvim^68^ points out that the main source of phosphorus for the Piabanha River is the sewage discharge and the higher concentrations are found during the rainy season.

Nitrate is a very common element in surface water since it is the end product of the aerobic decomposition of the organic nitrogenous compound^70,71^. Its sources are related to landscape composition, being influenced by both agricultural and urban uses^72^. Villas-Boas^42^ found high concentration of nitrate and ammonium in the urban region of Piabanha River in agreement with this study. Alvim^68^ reports that domestic sewage discharged into Piabanha River waters account for 43% of the nitrogen load, the atmospheric contribution for 31% and the farming activity for 15%.

### The major contributors to water quality and stretches of river with similar water quality

The first two components together account for 86% of the total variance, indicating method high explanatory power of the method. It was far better than other similar studies around the world^29,30,71,73–75^. PC1 predominantly accounts for urban sewage pollution. This is clearly demonstrated by the fact that stations from 1 to 4, located in the urban area of Petrópolis, positively loaded PC1 with organic matter (BOD and COD), TDS and nutrients such as phosphorus and nitrogenous constituents, especially ammonia, indicating recent pollution. Even clearer is the fact that stations from 5 to 8 have negatively loaded with nitrate, showing the nitrogen compounds degradation in the downstream stretches of the urban area. On the other hand, the increase in nitrate concentrations in association with the increase in turbidity in stations outside the urban area may also be associated with land use, especially in agriculture.

PC2 is dominated by the dissolved oxygen parameter and other parameters that indicate the health of the river, as organic load and coliforms. It is explained by water pollution by organic matter and biological activity and reinforces the result of CP1. In the study region, sanitation is still a challenge to be faced by the government, especially in the first urban stretch, after 40 km from the source of the Piabanha River, this region has 26% of untreated sewage^53^.

Cluster analysis was used to group sampling stations into similarity classes indicating the stretches of river with similar water quality. As pointed out by Singh^29^, it implies that only one site in each cluster may serve as good in spatial assessment of the water quality as the whole cluster. So, the number of sampling sites can be reduced; hence, cost without losing any significance of the outcome. On the other hand, this interpretation should be done with caution since trends in different stretches can be very different, making future changes significant. Therefore, great care must be taken to reduce monitoring stations.

It is important to notice that the first cluster (S1, S6 and S4, S5) groups station 1 with station 6, the first one corresponding to the urban area of Petrópolis whose pollution stems from sewage and industrial effluents. Likewise, station 6 is located after the confluence of the Preto-Paquequer River, which crosses Teresópolis, the second largest city in the hydrographic basin, also with the presence of economic and industrial activities. Sand mining is the predominant activity near stations 4 and 5, which together receive the impact of five mining companies. Similarly, station 6, after the Preto River, receives the impact of seven sand mines. In fact, this group brings together economic activities whose impact on water quality is similar. Station 5 could be removed from the network monitoring in order to reduce costs.

The second cluster (S2 and S3) refers to the most urbanized section of the basin. When individually checking the quality parameters between these stations, one can conclude that they differ only by the diluting effect caused by the contribution of the Araras River, on the left bank, and of the Poço do Ferreira River, on the right bank, which receives its waters from the Bonfim River after its source in the Serra dos Órgãos National Park, an important federal conservation unit. Station 3 was introduced precisely to detect this diluting effect, but since the cluster analysis showed that it was not significant it is recommended to remove this station.

The third cluster (S7 and S8) has a very similar behavior: station 8 is just before the Piabanha River mouth and station 7 is located less than 10 km upstream of the mouth. In addition, on this stretch there are only three interferences registered as discharges. Thus, it is recommended to remove station 7, considering the importance of maintaining a station close to the river mouth.

### Trend analysis and seasonal variation

Although it still presents systematic violations to Brazilian standards^76^, the water quality, in general, has improved in the Piabanha River over the past 40 years (Fig. 3A,B). This statement is supported by the Mann–Kendall rank test of randomness, indicating a significant (p = 0.03) tendency to increase the values of the dissolved oxygen parameter at station PB002, located in the urban area of Petrópolis, which is highly impacted by effluent discharges, despite the fact that this region has municipal sewage treatment. PB011 presents high levels of DO, since the beginning of the time series exhibiting an almost monotonic behavior over time, thus it has no tendency. The high DO levels are due to both the river's reoxygenation process and the contribution of clean waters from its tributaries, such as the Fagundes River.

A strong annual and semi-annual seasonality was indicated by the power spectral density, which can be seen in the periodogram (Fig. 3B) resulting from the Fast Fourier Transform. The results are in accordance with the literature^77^ indicating that more than 90% of the total variance of dissolved oxygen is accounted for by the annual periodicity and the next four higher harmonics (semi-annual; tri-annual, etc.). Seasonality follows the rainfall regime with a dry period from April to September, and a wet period from October to March, according to Araújo's^78^ study carried out in the Piabanha River basin.

Water quality at point PB002 started to improve in 2000, when the first sewage treatment plant in the city of Petrópolis came into operation. Currently, 95% of the population has access to drinking water, and the coverage of treated urban sewage is 85%. The municipality has 26 sewage treatment units, responsible for the treatment of 56.2 million liters per day. In relation to the other municipalities in the basin, according to the National Sanitation Information System^79^ (SNIS), the municipality of Três Rios treats 2.97% of its sewage, while the other municipalities, Teresópolis, Areal, São José do Vale do Rio Preto, Paty do Alferes and Paraíba do Sul did not report their data to SNIS, potentially indicating that they do not perform sewage treatment. In other words, about 50% of the population has no formal access to sewage treatment services.

## Conclusion

The diagnosis provided by this research establishes the first step towards the Framing of water resources according to their intended uses, as established by the Brazilian National Water Resources Policy. In addition to the diagnosis which was carried out a georeferenced database was built. There are few cases of Framework in Brazil and none in the studied watershed. This makes this study relevant to Brazilian water resources management. The considerable number of users awaiting regularization from the State Environmental Institute is a limitation to implement the Framework and requires a joint effort of the watershed committee.

Answering our initial question, Piabanha River water quality is medium according to the WQI_NSF_ and certainly is not able to support high levels of biodiversity. Some river stretches have quality compatible with class 4 according to the Brazilian regulation for the coliforms, BOD and TP parameters; hence, they cannot be used for irrigation, human or animal consumption, not even after treatment. On the other hand, the Framework must be carried out according to intended uses. Therefore, we recommend that the Piabanha Committee, in partnership with the State Public Ministry, lead actions to reduce the concentrations of these parameters, mainly in the sanitation sector.

It is recommended that the monitoring program be continued and expanded to stretches where conflicts between water uses occur, in order to implement the Framework to enforce the improvement of water quality. It is also important to point out that this study was financed with public resources from the Piabanha water resources fund and that the present analysis made possible to recommend the exclusion of three of the eight existing stations, thereby enabling the expansion of the monitoring to other tributaries of the Piabanha River under the influence of large population with practically no sanitation, notably the Rio Preto/Paquequer sub-basin.

This work describes a methodological approach that can be useful for other researches in environmental science and management. We have applied an integrated approach using data from different sources combined with data analysis based on WQI, PCA, CA, frequency analysis and trend analysis, which were used in a complementary way to understand a research problem.

## Materials and methods

### Study area

**Figure 4:**
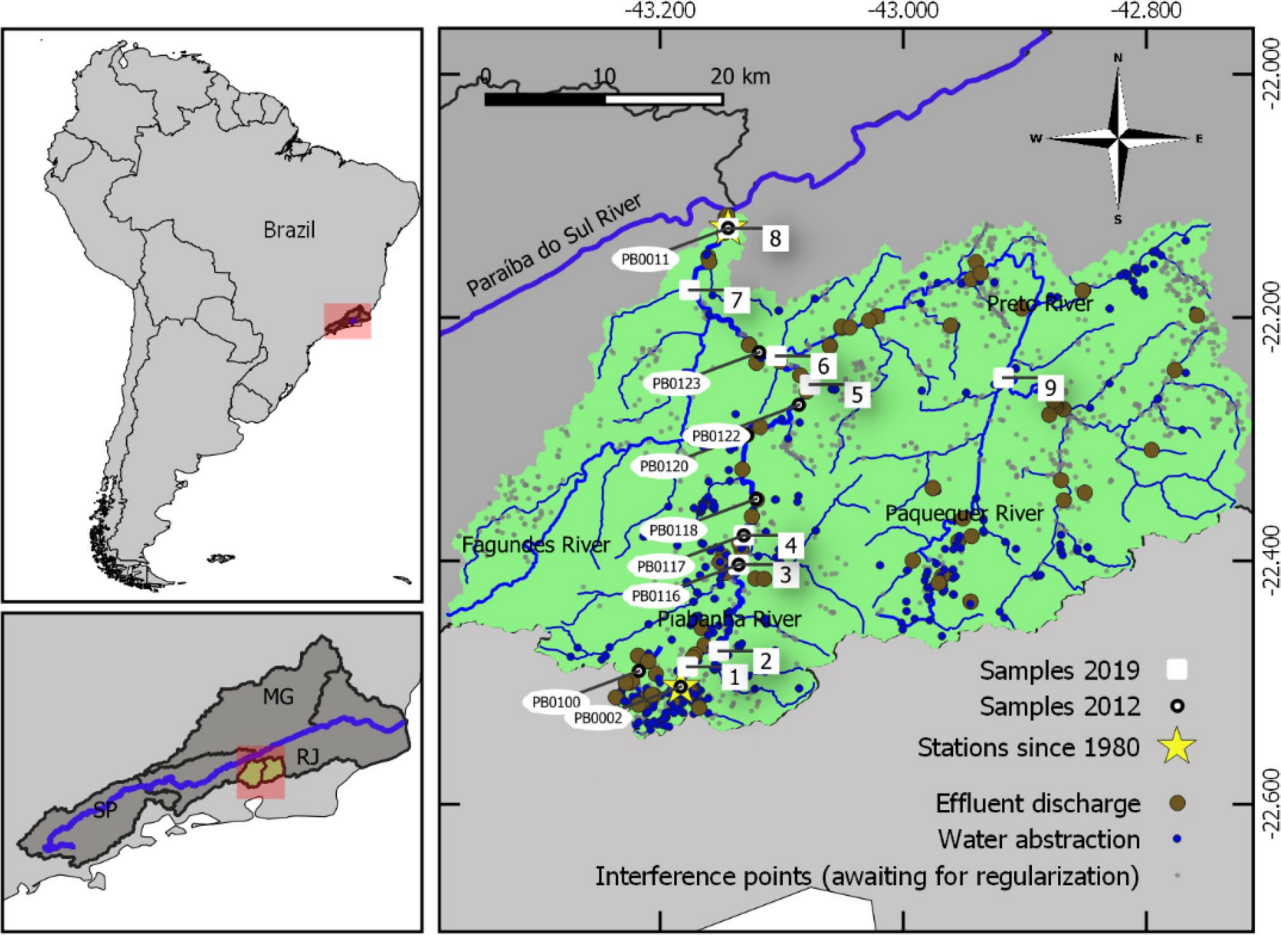
Study area, sample stations and interference points (water abstraction or effluxent discharge). This map was generated in the open source software QGIS version 3.14.15 (https://qgis.org/).

The Piabanha Basin is located in southern Brazil, belonging to the mountainous region of the State of Rio de Janeiro with an area of 2050 km^2^ (Fig. 4). The Piabanha River source is at 1150 m of altitude and runs down 80 km until it flows into the Paraíba do Sul River at an altitude of 260 m. The upper portion of the basin presents a humid tropical climate. With steep slopes, annual rainfall exceeds 2000 mm. The lower portion of the basin has a sub-humid climate and the average rainfall decreases to 1300 mm. The seasons are well defined throughout the basin and the rainfall regime has symmetry in its distribution between the periods from January to June and from July to December^78^. The territory is home to 535 thousand people in 2018^80^. The two largest cities in the region, Petrópolis and Teresópolis, are located in the headwaters of the basins and give rise to the Piabanha and Preto rivers, respectively. Additionally, because the sewage treatment is limited and the river flows are low, high constituent concentrations are observed (e.g., fecal coliform, nitrate, and BOD), especially in urban areas^42^.

### Datasets

Three sets of monitoring data have been used in this researchh (Fig. 4). The first and main one was the result of a monitoring program that is being conducted by the Piabanha watershed Committee, in which data from July to December 2019 have been analysed and are described in more details in the next item. The second were from 6 campaigns carried out in 2012 by HIDROECO project^44^ also with financial resources from the Piabanha Committee which is used as a baseline for comparison purposes. The third was comprised of two stations of the basic monitoring network of the Rio de Janeiro Environmental Institute, with data from 1980 to the present, except for periods of data gaps.

A georeferenced database was also built containing water management data. Brazilian National Water Agency (ANA) has developed the National Water Resources Users Register (CNARH) for any bulk water user that changes regime, quantity or quality of a water body. It is a federal platform, but it can be managed by each state. Registration is a prerequisite for the other stages of uses regularization.

### Monitoring campaigns and analytical procedures

Physical–chemical parameters were measured in situ using a multiparameter probe (YSI model 556) and a portable turbidimeter (HANNA model HI 98703-0), both previously calibrated and later verified. The samples were placed in specific containers for each analysis, for the necessary parameters the samples were preserved with H_2_SO_4_ and kept at a temperature below 4 °C. Laboratory analyses (Table 1) were performed according to Standard Methods for the Examination of Water and Wastewater (SMWW)^81^. The laboratory has an accreditation certificate issued by the State Environmental Agency (INEA CCL No. IN044710) and also complies to ISO/IEC 17025 (CRL 1035).

### Water Quality Index

A Water Quality Index (WQI) is an empirical expression which integrates significant physical, chemical and microbiological parameters of water quality into a single number^82^. It can be a powerful communication tool to simplify a complex set of parameters, whose individual interpretation can be difficult, into a single index representing the general water quality. A water quality index was initially proposed by Horton^26^ and further developed by Brown^27,83^ resulting in the National (USA) Sanitation Foundation Water Quality Index (WQI_NSF_).

The original version of the WQI_NSF_ established an additive expression^27^; on the other hand, field data analysis suggested that the additive WQI lacked sensitivity in adequately reflecting the effect of a single low value parameter on the overall water quality. As a result, a multiplicative form of WQI was proposed^82,83^:

$$WQI_{NSF} = \mathop \prod \limits_{i = 1}^{n} q_{i}^{{w_{i} }}$$

*q*_*i*_ is the quality class for the *n*th variable, a number between 0 and 100, obtained from the respective average quality variation curve^82^, depending on the concentration of each nth variable. *W*_*i*_ is the relative weight for the *n*th variable, number between 0 and 1, assigned according to the importance of the variable for overall quality conformation. WQI_NSF_ is the National Sanitation Foundation Water Quality Index, a number between 0 and 100, rated as "excellent" (100 > WQI ≥ 90), "good," (90 > WQI ≥ 70), "medium" (70 > WQI ≥ 50), "bad" (50 > WQI ≥ 25) or "very bad" (25 > WQI ≥ 0).

The WQI_NSF_ and its many adaptations have been widely used^84,85^, however, its use is not uniform, replacing parameters without the necessary adaptation of the respective curve of the indicator. In Brazil, since 1975 the WQI_NSF_ has been used by CETESB (Environmental Company of the State of São Paulo). In the following decades, other Brazilian states adopted, with minor adaptations, this index, which today is the most widely used in the country. In the present study, the weights (w_i_) have been used according to the methodology established by INEA (Environmental Institute of the State of Rio de Janeiro): DO (0.17); Fecal coliforms (0.16); pH and BOD (0.11); Nitrates, Phosphate and Temperature (0.10); Turbidity (0.08) and TDS (0.07), rather than total solids.

The replacement of the total solids for dissolved solids parameter may cause an average variation of 0.2% in the final result of WQI_NSF_, based on our estimates (n = 48, data 2019). In relation to microbiology, *E. coli* have been used instead of fecal coliforms, applying a correction factor^86^ of 1.25 on the result of *E. coli*.

### Principal component analysis and cluster analysis

Principal component analysis (PCA), as defined by Hotelling^87^, is a multivariate technique of covariance modeling that reduces the dimensionality of an originally correlated dataset, with the lowest possible information loss. A new set of variables containing new orthogonal, uncorrelated variables, is formed from a dataset of correlated variables, which are weighed linear combinations of the original variables^30^.

PCA technique extracts the eigenvalues and eigenvectors from the covariance matrix of original variables. The PCs are obtained by multiplying the original correlated variables with the eigenvector, which is a list of coefficients, frequently called “loadings”^29,30,88,89^. A widely accepted and simple qualitative rule proposes that loadings greater than 0.30 or less than − 0.30 are significant; loadings greater than 0.40 or less than − 0.40 are more important, whereas loadings greater than 0.50 or less than − 0.50 are very significant^90^. The suitability of data for PCA was evaluated by Kaiser–Meyer–Olkin^91,92^ (KMO) measuring of sampling adequacy and Bartlett tests of sphericity^93^. The Shapiro test was evaluated to verify the data normality (α = 0.01).

Cluster analysis reveals the latent behavior of a dataset to categorize the objects into groups or clusters on the basis of similarities^30,88,89^. Hierarchical agglomerative cluster analysis (CA) classifies objects by first putting each object in a separate cluster, and then joins the clusters together stepwise until a single cluster remains^29^.

### Timeseries analysis and trend detection

Mann–Kendall trend test is a nonparametric test used to identify a trend in a series, first proposed by Mann^94^ and further improved by Kendall^95^ and Hirsch^96^. The null hypothesis (H_0_) for these tests is that there is no trend in the series. The tests are based on the calculation of Kendall's tau measure of association between two samples, which is itself based on the ranks with the samples. The variables are ranked in pairs, and the difference of each variable to its antecessor is calculated. The total number of pairs that present negative differences is subtracted from the number of pairs with positive differences (S). A positive value of S indicates an upward trend, and a negative value of S a downward trend. For n > 10, a normal approximation is used to calculate Z statistic which is used to calculate p-value^96^.

Fourier decomposition is a technique which allows the separation of frequency components from a data series with seasonal behavior from a complex water quality dataset^97^. Spectral analysis performed using a Fast Fourier Transform (FFT) algorithm is widely used in environmental studies, because it reveals the dominant influences and their scales^50^. Power spectral density (PSD) obtained from FFT and represented by periodograms is a recommended procedure to detect seasonality^98,99^.

### Brazilian legal regulation

Brazilian fresh waters are divided into four classes, depending on the intended use^76^. The Special Class is intended mainly for the preservation of the natural balance of aquatic communities in fully protected conservation areas. Class 1 is designed for human consumption supply, after simplified treatment, for the protection of aquatic communities and for primary contact recreation. Class 2 requires conventional treatment for human consumption. Class 3 requires conventional or advanced treatment for human consumption and can be used to feed animals and irrigate some crops. Class 4 is intended only for navigation and landscape harmony. It is important to note that the Framework refers to the required water quality target according to water uses. The river basin committees are responsible for implementing the Framework, in accordance with the Brazilian National Water Resources Policy^33^. As long as the Framework is not established by the basin committee, fresh waters will be considered class 2 (Art. 42 CONAMA 357/2005)^76^.

## Supplementary Information

Supplementary Table S1.

Supplementary Table S2.

Supplementary Table S3.

Supplementary Table S4.

Supplementary Table S5.

## Data Availability

All data generated or analysed during this study are included in this published article and its Supplementary Information files.
